# Regulation of the photophysical dynamics of metal nanoclusters by manipulating single-point defects

**DOI:** 10.1038/s41467-025-65024-3

**Published:** 2025-11-17

**Authors:** Peiyao Pan, Weinan Dong, Wentao Huang, Xue Bai, Zhennan Wu, Xi Kang, Manzhou Zhu

**Affiliations:** 1 Key Laboratory of Structure and Functional Regulation of Hybrid Materials of Ministry of Education, Anhui Province Key Laboratory of Chemistry for Inorganic/Organic Hybrid Functionalized Materials, Department of Chemistry, Anhui University, Hefei, China; 2 State Key Laboratory of Integrated Optoelectronics, JLU Region, College of Electronic Science and Engineering, Jilin University, Changchun, China; 3 National Key Laboratory of Opto-Electronic Information Acquisition and Protection Technology, Anhui University, Hefei, Anhui China

**Keywords:** Inorganic chemistry, Organometallic chemistry

## Abstract

Metal nanoclusters have served as an emerging class of programmable nanomaterials with customized structures. However, it remains highly challenging to achieve the single-atom regulation of metal nanoclusters without altering their structural frameworks. Here, we achieve the single-point defects manipulation based upon a cluster pair of Au_21_ and Au_22_ by meticulously complementing the surface defects of the former nanocluster with an additional single-Au complex. The two nanoclusters exhibited identical geometric structures, but their pronounced quantum-confinement effects resulted in different electronic properties, evident in their distinct optical absorption and emission characteristics. Temperature-dependent steady-state photoluminescence spectra and femtosecond transient absorption spectra showed that the manipulation of a single-point defect in Au_22_ inhibited non-radiative decay pathways, reduced electron loss at higher energy levels, and accelerated intersystem crossing, which ultimately enhanced its emission intensity. Overall, the Au_21_ and Au_22_ cluster system in this study provides a cluster platform with controllable surface single-point defects, enabling the regulation of the photophysical dynamics at the atomic level.

## Abstract (web summary)

The properties of atomically precise nanoclusters depend on their structural composition, but controlling them is challenging. Here, the authors demonstrate the manipulation of single-point defects, converting Au_21_ into Au_22_ and thereby regulating the photophysical dynamics.

## Introduction

Nanoscience has been flourishing since Richard Feynman’s groundbreaking speech about the possibilities of manipulating matter at the atomic level, famously titled “There’s Plenty of Room at the Bottom”^1,2^. For a long time, it has been the dream of nanoscientists to master atomic-level manipulations and control the structure of nanomaterials precisely. With the ongoing accumulation of synthetic knowledge and the development of advanced analytical methods, researchers can now tailor the composition and morphology of metal nanoparticles^3–6^. However, achieving atomic-level adjustments at specific sites on the nanoparticle surface—such as adding or removing one or two metal atoms at designated positions-remains challenging. These atomic modifications are crucial, as they control the physicochemical properties of the nanomaterials^7–11^.

Metal nanoclusters are an emerging class of promising nanomaterials due to their atomically precise structures^12,13^. Their nanoscale sizes endowed these nanoclusters with molecular-like characteristics, featuring discrete electronic energy levels and strong quantum-confinement effects^14–16^. Indeed, the quantum-confinement effects of metal nanoclusters render them programmable nanomaterials with structure-dependent properties, and any perturbations on compositions/structures may induce variations in clusters’ physicochemical performances^5,17–19^. In turn, the atomically precise nature of nanoclusters enables researchers to master the structure-property correlations, which is essential for visualizing the quantum-confinement effects of these nanomaterials^20–23^. In this context, it is necessary to develop structurally analogous cluster systems with comparable properties for such a visualization, which requires the atomic-level manipulation of nanoclusters^24–30^. However, as of now, it remains highly challenging to achieve the single-atom regulation of metal nanoclusters without altering their structural frameworks^31–35^. Atomic-level understanding of the structure-dependent physical-chemical properties requires newly developed cluster systems as model platforms and precise tools^36–43^.

Herein, the atomic-level manipulation has been accomplished using two structurally analogous gold cluster molecules, [Au_21_(AdmS)_12_(PPh_2_py)_3_]^+^ and [Au_22_(AdmS)_12_(PPh_2_py)_4_]^2+^ (abbreviated to Au_21_ and Au_22_, respectively), with which the photophysical dynamics of metal nanoclusters have been regulated at the atomic level. Specifically, the single-point defect of the Au_21_ nanocluster could be complemented by the addition of a single-gold-atom complex, giving rise to its structural analog, the Au_22_ nanocluster. The maintained structural framework and the single-atom disparity of the two nanoclusters rendered them a platform for visualizing the quantum-confinement effects in determining their photophysical properties. While both cluster analogs maintained a consistent geometric framework, they exhibited evidently different electronic structures and distinct chromatic properties―Au_21_ displayed reddish-brown absorption, whereas Au_22_ showed yellowish-green absorption. Additionally, the Au_22_ showed much brighter photoluminescence (PL) compared to the single-point defective Au_21_, with PL quantum yields of 47.63% for Au_22_ and 13.10% for Au_21_. Such differences in photophysical properties, triggered by the single-atom manipulation, have been unambiguously rationalized using a combination of temperature-dependent steady-state PL spectroscopy and femtosecond transient absorption (fs-TA) spectroscopy. Our findings revealed that the weaker electron-phonon coupling and faster intersystem crossing (ISC) in Au_22_ contributed to its enhanced emission intensity.

## Results

### Synthesis and structural characterization

The Au_21_ nanocluster was synthesized using a one-pot synthetic method by directly reducing Au-AdmS-PPh_2_py complexes with NaBH_4_ (Supplementary Fig. 1a; see Methods). The CH_2_Cl_2_ solution of Au_21_ was reddish-brown; however, upon the addition of the AuPPh_2_pyCl complex to Au_21_, the solution color altered from reddish-brown to yellowish-green, indicating the cluster transformation from Au_21_ to Au_22_, derived from their mass characterizations (Supplementary Fig. 1b,c). To assist the sample ionization in electrospray ionization mass spectrometry (ESI-MS), cesium acetate (CsOAc) was added to the cluster sample. As shown in Supplementary Figs. 1c, 2, two prominent mass signals at *m/z* of 3549.67 and 3696.46 were detected in the positive mode, which matched well with the chemical formulas of [Au_21_(AdmS)_12_(PPh_2_py)_3_(CH_3_OH)Cs^+^]^2+^ (calc *m/z* 3549.68) and [Au_22_(AdmS)_12_(PPh_2_py)_4_]^2+^ (calc *m/z* 3696.34), respectively. In this context, the Au_21_ and Au_22_ nanocluster molecules were in “+1” and “+2”-charge states, respectively, demonstrating their identical free valence electron numbers of 8e, i.e., 21(Au) − 12(SR) – 1(charge state) = 8 for the Au_22_ nanocluster and 22(Au) – 12(SR) – 2(charge state) = 8 for Au_21_.

**Fig. 1:**
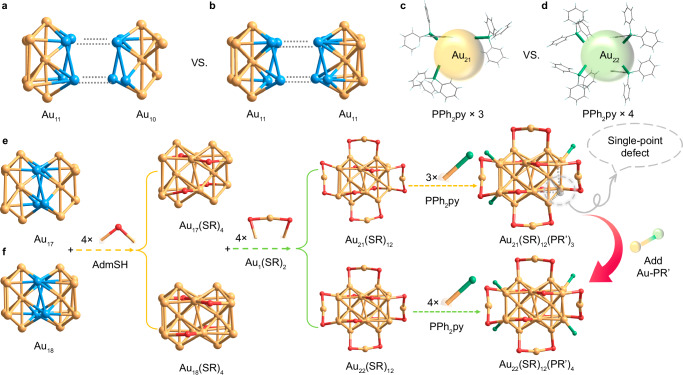
Structural comparison between Au_21_ and Au_22_ nanoclusters. **a** The Au_17_ kernel of the Au_21_ nanocluster comprises one Au_11_ and one Au_10_ units by sharing four Au atoms. **b** The Au_18_ kernel of the Au_22_ nanocluster consists of two Au_11_ units by sharing four Au atoms. **c** Three PPh_2_py ligands acting as terminals of the Au_17_ core. **d** Four PPh_2_py ligands acting as terminals of the Au_18_ core. **e** Structural anatomy of the Au_21_ nanocluster with a peripheral single-atom defect. **f** Structural anatomy of the Au_22_ nanocluster with a full-protected surface. SR AdmS; PR’ PPh_2_py. Color legends: light blue sphere and orange sphere, Au; red sphere, S; green sphere, P; light grey sphere, N; grey sphere, C; white sphere, H.

Single crystals of Au_21_ and Au_22_ nanoclusters were cultivated by diffusing hexane into their CH_2_Cl_2_ solutions over 7 days, and their atomically precise structures were determined using single-crystal X-ray diffraction (Supplementary Tables 1,2). The Au_21_ cluster crystallized in the orthorhombic space group *Fddd*, while Au_22_ crystallized in a monoclinic system with a *C2/c* space group, resulting in distinct packing arrangements within their respective crystal lattices. Structurally, the two nanoclusters exhibited comparably geometric structures while the surface single-point defect of the Au_21_ nanocluster was complemented by a single-gold-atom complex, giving rise to its structural analog, the Au_22_ nanocluster (Fig. 1). Specifically, the Au_17_ core of the Au_21_ nanocluster could be conceptualized as consisting of a twisted Au_11_ unit and a twisted Au_10_ unit that share an Au_4_ face (Fig. 1a). In contrast, the Au_18_ core of the Au_22_ nanocluster was made up of two twisted Au_11_ units by sharing the same Au_4_ face (Fig. 1b). While the Au_10_/Au_11_ structures here adopted a cuboctahedral shape, their cuboctahedral configuration was distorted. Additionally, the overall structure of Au_21_, depicted in Fig. 1c,e, featured an Au_17_ kernel protected by four Au_1_(SR)_2_ motifs, four μ_2_-SR ligands, and three vertex PPh_2_py ligands. In comparison, the Au_22_ nanocluster contained the same surface Au_1_(SR)_2_ and μ_2_-SR stabilizers as those found in Au_21_, while four PPh_2_py ligands were anchored at vertex positions of the Au_18_ kernel (Fig. 1d,f). Collectively, the introduced single gold atom would not alter the overall framework of the Au_21_ nanocluster but fill in its surface defects by anchoring an additional PPh_2_py stabilizer, giving rise to a structurally analogous nanocluster pair with single-point defects manipulation.

Although the introduction of a single gold atom to the surface defect of Au_21_ would not alter its overall skeleton, the corresponding bond lengths underwent readjustment. As depicted in Supplementary Fig. 3a, the average Au-Au bond length of the Au_18_ kernel in Au_22_ was shorter than that of the Au_17_ kernel in Au_21_, measuring 2.899 Å compared to 2.953 Å. Moreover, the single-point defect in Au_22_ caused a notable decrease in both peripheral Au-P and Au-S bond lengths (Supplementary Fig. 3b,c). In this context, at the molecular level, the introduction of a surface single-gold-atom rendered the cluster skeleton more compact, and thus the overall framework of Au_22_ was more rigid. The comparisons of the metal skeleton and the core size of the two clusters also support this perspective. As shown in Supplementary Fig. 4, the length and width of the Au_22_ nanocluster are 9.87 and 10.03 Å, respectively, both shorter than those of the Au_21_ nanocluster. Additionally, the core of the Au_22_ cluster is more compact than that of the Au_21_ cluster. The strengthened structural rigidity of the Au_22_ nanocluster might enhance the emission intensity of nanoclusters in their molecular states by minimizing vibrational relaxation (discussed below)^44,45^.

From a supramolecular chemistry perspective, the crystalline packing arrangement of Au_21_ and Au_22_ nanoclusters in the lattice differed significantly. The intercluster distances between adjacent Au_21_ molecules were determined as 20.11 and 22.08 Å, while those for Au_22_ were 17.98 and 19.80 Å, demonstrating that the Au_22_ cluster molecules were more closely packed in the crystal lattice (Supplementary Figs. 5–7). In addition, several intermolecular hydrogen interactions (H∙∙∙H) were observed between adjacent Au_21_ nanoclusters with an average interaction length of 2.32 Å (Supplementary Fig. 8a,b). By comparison, the crystal lattice of Au_22_ not only contained several intermolecular H∙∙∙H interactions (average interaction length: 2.43 Å) but also included multiple C-H∙∙∙*π* interactions (Supplementary Fig. 8c-e). Such rich intermolecular interactions were probably responsible for the more closely packed Au_22_ clusters in the crystal lattice^46,47^.

### Photophysical properties

**Fig. 2:**
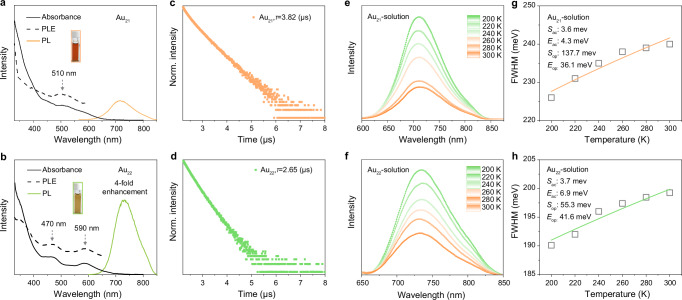
Optical analysis. **a** Optical absorptions, excitations (PLE), and emissions (PL) of Au_21_ nanoclusters. **b** Optical absorptions, excitations (PLE), and emissions (PL) of Au_22_ nanoclusters. **c** PL lifetimes of Au_21_ nanoclusters. **d** PL lifetimes of Au_22_ nanoclusters. **e** Temperature-dependent PL spectra of Au_21_ nanoclusters dissolved in CH_2_Cl_2_. **f** Temperature-dependent PL spectra of Au_21_ and of Au_22_ nanoclusters dissolved in CH_2_Cl_2_. **g** FWHM of the steady-state PL spectra as a function of temperature for Au_21_ nanoclusters dissolved in CH_2_Cl_2_. **h** FWHM of the steady-state PL spectra as a function of temperature for Au_22_ nanoclusters dissolved in CH_2_Cl_2_.

Due to the strong quantum-confinement effect of metal nanoclusters with nanoscale sizes, the single-point defect manipulation would result in differences in the photophysical properties of the Au_21_/Au_22_ cluster pair. Indeed, the single-atom regulation has been shown to affect the geometric structures of the two nanoclusters, which would ultimately influence their electronic structures and optical performances. As illustrated in Fig. 2a,b, the CH_2_Cl_2_ solution of Au_22_ displayed intense optical absorption around 590 nm, accompanied by a shoulder band at 470 nm, whereas Au_21_ presented only a weak and broad peak at 510 nm. The PL properties of Au_21_ and Au_22_ nanoclusters were then evaluated under ambient conditions. As shown in Fig. 2a,b, strong emissions of Au_21_ and Au_22_ nanoclusters occurred at 715 and 730 nm, respectively, when the cluster solution was excited at 375 nm. The PL intensity of Au_22_ was four times greater than that of Au_21_. In addition, the PL QY of the Au_22_ nanocluster was determined to be 47.63%, evidently enhanced from the 13.10% of the Au_21_ nanocluster with a surface single-point defect. The Au_22_ nanocluster exhibited a refined structure symmetry relative to Au_21_, which improved the structure rigidity and weakened the framework vibration of the former cluster, resulting in its higher PL intensity. Furthermore, the average PL lifetimes of Au_21_ and Au_22_ were measured as 2.65 and 3.82 μs, respectively, and the microsecond lifetimes suggested their analogous phosphorescent characteristic (Fig. 2c,d). To obtain more accurate and direct measurements, we tested the excitation spectra of the two clusters and found a high degree of agreement with their corresponding absorption spectra (Fig. 2a,b; see Methods for test conditions). As shown in Supplementary Fig. 9, the excitation-dependent emission spectra indicate single sources of Au_21_ and Au_22_. In this context, the characteristic peaks of the excitation spectra aligned with the absorption spectra of the two nanoclusters, suggesting that the absorption and emission processes occur at the same energy level^48,49^.

To investigate the effect of single-point defect manipulation on photophysical and vibration properties of the two gold nanoclusters, we further analyzed their temperature-dependent steady-state PL spectroscopy. A consistent increase in PL intensity was observed for both nanoclusters in their solution state as the temperature decreased from 300 to 200 K (Fig. 2e,f). To better understand the vibrational properties of Au_21_ and Au_22_ nanoclusters, we extracted the temperature-dependent full width at half maxima (FWHM) of their emission peaks, and the plots are given in Fig. 2g,h. The distribution of FWHMs could be well fitted according to Eq. (1)^50–52^.

1 $$\varGamma \left(T\right)={\varGamma }_{0}+\sqrt{{{S}_{{{{\rm{ac}}}}}{E}_{{{{\rm{ac}}}}}\coth \left(\frac{{E}_{{{{\rm{ac}}}}}}{2{{{{\rm{k}}}}}_{{{{\rm{B}}}}}T}\right)}+S_{{{{\rm{op}}}}}{E}_{{{{\rm{op}}}}}\frac{1}{{{{{\rm{e}}}}}^{\frac{{E}_{{{{\rm{op}}}}}}{{{{{\rm{k}}}}}_{{{{\rm{B}}}}}T}}-1}}$$

Upon where *Γ*_0_ was the temperature-independent intrinsic linewidth, Sac and Sop were the coupling strengths for acoustic phonons and optical phonons, respectively, and *E*_ac_ and *E*_op_ were the average energy of acoustic phonons and optical phonons. As illustrated in Fig. 2e–h, the core-directed low-frequency acoustic phonons (4.3 meV for Au_21_ and 6.9 meV for Au_22_) barely affected the cluster emission, while the fitted optical phonon energies (36.1 meV for Au_21_ and 41.6 meV for Au_22_) indicated that the Au-S vibrations from cluster surfaces or interfaces dominated their non-radiation. The coupling strength of 55.3 meV for Au_22_ was crucially lower than the value of 137.7 meV for Au_21_, suggesting a weaker electron-phonon coupling and less PL quenching of the former nanocluster.

### Electron dynamics

**Fig. 3:**
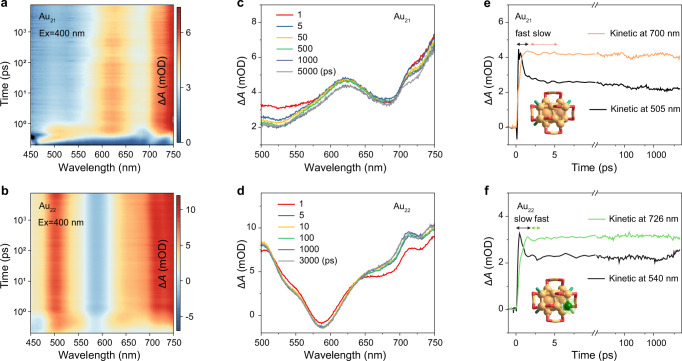
Excited state dynamics of Au_21_ and Au_22_ nanoclusters dissolved in CH_2_Cl_2_. Data map of femtosecond TA of **a** Au_21_ and **b** Au_22_ nanoclusters pumped at 400 nm. TA spectra of **c** Au_21_ and **d** Au_22_ nanoclusters at different time delays. TA kinetic traces selected at specific probe wavelengths of **e** Au_21_ and **f** Au_22_ nanoclusters.

For molecular-state metal nanoclusters, their PL QYs depend not only on the electron transition of the luminescent state but also on the electron relaxation process in the upper energy levels^53,54^. Femtosecond transient absorption (fs-TA) spectroscopy was then performed to trace the electron trajectory before reaching the luminescent state. Upon the 400 nm excitation and the 500–750 nm detection, two distinct ground-state bleaching (GSB) dents near 520 and 670 nm were obtained in the 2D-TA map for Au_21_ (Fig. 3a,c). Given the total positive signal distribution, the very broad excited-state absorption (ESA) should span the entire probe region, and the differences between absorption and GSB peak positions should arise from the ESA modification. For Au_22_, a main GSB band at 590 nm overlapped with broad ESA was observed, which precisely corresponded to the main absorption peak in steady state, thus reflecting its structure integrity during the measurement (Fig. 3b,d). Of note, the TA signals of Au_21_ and Au_22_ underwent essential changes only for the initial few picoseconds, and then converged to a stable situation.

From the TA kinetic traces selected at specific probe wavelengths of Au_21_ and Au_22_ nanoclusters (Fig. 3e,f), we speculated that at the early part of the TA dynamics, the Au_21_ nanocluster first showed a faster electron injection process and followed by a slower electron decay process compared to Au_22_, finally an electron relaxation process exceeding the detector capacity. Global fitting required three decay components to fit the dynamics (0.36 ps, 2.61 ps, and > 1 ns for Au_21_ and 0.53 ps, 1.11 ps, and > 1 ns for Au_22_) (Supplementary Fig. 10a–d). The <1 ps dynamics could be explained as the internal conversion (IC) of hot electrons from S_*n*_ to the S_1_ state since the values were importantly reduced under the 530 nm pump (Supplementary Fig. 10e, f). Given the phosphorescent characteristic from the triplet state of the two nanoclusters, the few picoseconds were attributed to their intersystem crossing (ISC). The last >1 ns component accounted for the electron−hole recombination because of their μs-level luminescence lifetimes.

### PL mechanism

**Fig. 4:**
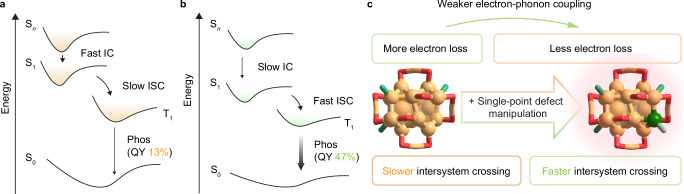
Comparison of PL mechanisms. PL mechanism of **a** Au_21_ and **b** Au_22_ nanoclusters. **c** Overview of the single-point defect manipulation towards electron-photon coupling and intersystem crossing of Au_21_ and Au_22_ nanoclusters. Color legends: green sphere and orange sphere, Au; red sphere, S; blue-green sphere and light grey sphere, P.

In this context, the PL mechanisms of Au_21_ and Au_22_ nanoclusters were proposed. Given the phosphorescence nature of Au_21_ and Au_22_, the excitation light should first pump the ground-state electrons to the excited singlet state, followed by a change in spin direction and eventually relaxed to the luminescent triplet state (Fig. 4a, b). Accordingly, the enhanced PL intensity of the Au_22_ nanocluster relative to Au_21_ could be rationalized from the following two aspects: (i) less energy loss in the upper energy levels, where the slower IC indicated more efficient electron relaxation of Au_22_; 2) faster ISC, which should arise from a reduced energy gap between singlet and triplet for Au_22_ through the single-point defect manipulation rather than the energy-level splitting induced by strong dipole-dipole interaction (Fig. 4c). Collectively, the single-point defect manipulation endowed the structurally comparable Au_21_ and Au_22_ nanoclusters with contrasting photophysical dynamics, and such differences originated from the strong quantum-confinement effects of such small gold nanoclusters.

## Discussion

In summary, the surface single-point defects of the Au_21_ nanocluster could be complemented by an additional single-Au complex, giving rise to its structural analog, the Au_22_ nanocluster with a maintained framework. Although the Au_21_ and Au_22_ nanoclusters exhibited nearly identical geometric structures, their electronic structures were significantly different due to strong quantum-confinement effects. The two nanoclusters manifested distinct photophysical properties, particularly in their optical absorption and emission characteristics. Such differences were rationalized by analyzing their temperature-dependent steady-state PL spectra and femtosecond transient absorption spectra. The single-point defects manipulation on Au_22_ inhibited the non-radiative decay pathways, reduced the electron loss at elevated energy levels, accelerated intersystem crossing, and ultimately enhanced its PL intensity. Collectively, the Au_21_ and Au_22_ cluster system, featuring a controllable single-point defect, provides a platform for visualizing single-atom manipulation effects in determining the photophysical dynamics of metal nanoclusters.

## Methods

### Materials

Adm-SH was prepared following a method reported in ref. ^55^. All following reagents were purchased from Sigma-Aldrich and used without further purification, including tetrachloroauric(III) acid (HAuCl_4_·3H_2_O, 99% metal basis), diphenyl-2-pyridylphosphine (PPh_2_py), sodium borohydride (NaBH_4_, 99%), methanol (HPLC grade), ethanol (HPLC grade), dichloromethane (HPLC grade), hexane (HPLC grade), and ethyl ether (HPLC grade).

### Synthesis of [Au_21_(AdmS)_12_(PPh_2_py)_3_]^+^

300 µL of HAuCl_4_·3H_2_O (0.2 g mL^−1^) and 50 mg of PPh_2_py were added into a mixed solvent of 10 mL of CH_3_OH and 10 mL of CH_2_Cl_2_, and the solution was stirred vigorously. After 10 min, a freshly prepared solution of NaBH_4_ (20 mg in 2 mL of water) was added, and the solution color changed to black immediately. Subsequently, 50 mg of Adm-SH was introduced to the solution. The reaction was proceeded for 8 h, after which the mixture was centrifuged at 10,000 × *g* for 5 min. The supernatant was collected and evaporated to yield the crude product, which was purified three times with CH_3_OH. Finally, the precipitate (insoluble in CH_3_OH) was dissolved in CH_2_Cl_2_, giving rise to the solution of the Au_21_ nanocluster, which was used directly in the crystallization process. The yield is 10% based on the Au element (calculated from the HAuCl_4_·3H_2_O) for synthesizing the Au_21_ nanocluster.

### Synthesis of [Au_22_(AdmS)_12_(Ph_2_py)_4_]^2+^

10 mg of the Au_21_ nanocluster was dissolved in a 20 mL of CH_2_Cl_2_, and 1 mg of AuPPh_2_pyCl complex was added. The solution color changed from red to green within 30 s, indicating the transformation from Au_21_ to Au_22_. The product was purified three times with CH_3_OH. Finally, the precipitate (insoluble in CH_3_OH) was dissolved in CH_2_Cl_2_, giving rise to the solution of the Au_22_ nanocluster, which was used directly in the crystallization process. The yield is 86% based on the Au element (calculated from the Au_21_ nanocluster) for synthesizing the Au_22_ nanocluster.

#### Crystallization of [Au_21_(AdmS)_12_(PPh_2_py)_3_]^+^ and [Au_22_(AdmS)_12_(PPh_2_py)_4_]^2+^

Single crystals of Au_21_ or Au_22_ nanoclusters were cultivated at room temperature by liquid diffusion of *n*-hexane into a CH_2_Cl_2_ solution containing the Au_21_ or Au_22_ nanocluster. After 7 days, red block crystals for Au_21_ based on Au and black block crystals for Au_22_ were collected, and the structures of the Au_21_ and Au_22_ nanocluster were determined.

#### X-ray crystallography

The data collection for single-crystal X-ray diffraction (SC-XRD) of all nanocluster crystal samples was carried out on a Stoe Stadivari diffractometer under nitrogen flow using a graphite-monochromatized Cu *K*_α_ radiation source (*λ* = 1.54186 Å). Data reductions and absorption corrections were performed using the SAINT and SADABS programs, respectively. The structure was solved by direct methods and refined with full-matrix least squares on F^2^ using the SHELXTL software package. All non-hydrogen atoms were refined anisotropically, and all hydrogen atoms were set in geometrically calculated positions and refined isotropically using a riding model. All crystal structures were treated with PLATON SQUEEZE, and the diffuse electron densities from residual solvent molecules were removed.

### Characterizations

Electrospray ionization mass spectrometry (ESI-MS) measurements were performed on a MicrOTOF-QIII high-resolution mass spectrometer. The sample was directly infused into the chamber at 5 μL min^−1^. For preparing the ESI samples, nanoclusters were dissolved in CH_2_Cl_2_ (1 mg mL^−1^) and diluted (*v*/*v* = 1:1) with CH_3_OH. All UV‒vis optical absorption spectra of the nanoclusters dissolved in CH_2_Cl_2_ were recorded using an Agilent 8453 diode array spectrometer, whose background correction was made using a CH_2_Cl_2_ blank. Nanocluster samples were dissolved in CH_2_Cl_2_ to make dilute solutions, followed by spectral measurement. Photoluminescence (PL) spectra were measured on an FL-4500 spectrofluorometer with the same optical density (OD) of ≈0.1. Absolute PL quantum yields (PL QYs) and emission lifetimes were measured with dilute solutions of nanoclusters on a HORIBA FluoroMax-4P. Femtosecond-TA spectroscopy was performed on a commercial Ti: Sapphire laser system (Spitfire SpectraPhysics; 100 fs, 3.5 mJ, 1 kHz). Solution samples in 1 mm path length cuvettes were excited by the tunable output of the OPA (pump). Excitation-dependent emission spectra involve adjusting the wavelength (*λ*_ex_) of the excitation light and recording the corresponding emission spectrum using an Edinburgh FLS1000 spectrofluorometer. Excitation spectra were conducted on the FL-4500 spectrofluorometer by fixing the emission wavelength and scanning the excitation wavelength.

### Reporting summary

Further information on research design is available in the Nature Portfolio Reporting Summary linked to this article.

## Supplementary information

Supplementary Information

Description of Additional Supplementary Files

Supplementary Data 1

Reporting Summary

Transparent Peer Review file

## Data Availability

The data that support the findings of this study are available from the corresponding authors upon request. Crystallographic data have been deposited at the Cambridge Crystallographic Data Centre (CCDC) under deposition numbers CCDC 2379695 (Au_21_) and 2379692 (Au_22_) and are provided as Supplementary Data 1.
