# Supplementary material for: Regulation of the photophysical dynamics of metal nanoclusters by manipulating single-point defects
Source: Nat Commun. 2025 Nov 17;16:10065. doi: 10.1038/s41467-025-65024-3 (PMC12623803; doi:10.1038/s41467-025-65024-3)
Supplement: Supplementary file 1 — Supplementary Information [file 41467_2025_65024_MOESM1_ESM.pdf]

Supplementary Information

Regulation of the photophysical dynamics of metal nanoclusters by manipulating single-point defects

Peiyao Pan,+[a] Weinan Dong,+[b] Wentao Huang,[a] Xue Bai,[b] Zhennan Wu,*[b] Xi Kang,*[a,c] Manzhou Zhu*[a,c]

[a] Key Laboratory of Structure and Functional Regulation of Hybrid Materials of Ministry of Education, Anhui Province Key Laboratory of Chemistry for Inorganic/Organic Hybrid Functionalized Materials, Department of Chemistry, Anhui University, Hefei 230601, P. R. China.

[b] State Key Laboratory of Integrated Optoelectronics, JLU Region, College of Electronic Science and Engineering, Jilin University, Changchun 130012, P. R. China.

[c] National Key Laboratory of Opto-Electronic Information Acquisition and Protection Technology, Anhui University, Hefei, Anhui 230601, P. R. China.

[+] These authors contributed equally.

*E-mails of corresponding authors: zmz@ahu.edu.cn (M.Z.); kangxi_chem@ahu.edu.cn (X.K.); wuzn@jlu.edu.cn (Z.W.)

This Supplementary Information file includes:

1. Positive-ion mode ESI-MS results of (a) Au_{21} and (b) Au_{22} nanoclusters.
2. Comparison of the bond lengths.
3. Comparison between overall structures of the two nanoclusters.
4. Packing of $[Au_{21}(AdmS)_{12}(PPh_2py)_3]^+$ nanocluster molecules in the crystal lattice from the a-axis, b-axis and c-axis.
5. Packing of $[Au_{22}(AdmS)_{12}(PPh_2py)_4]^{2+}$ nanocluster molecules in the crystal lattice from the a-axis, b-axis and c-axis.
6. Molecular spacing in the crystal lattice of (a) Au_{21} and (b) Au_{22} nanoclusters.
7. Illustration of the intercluster interactions in Au_{21} and Au_{22} nanoclusters.
8. Comparison of the excitation spectra and absorption spectra.
9. Decay-associated spectra (DAS) obtained from global fitting of (a) Au_{21} and (b) Au_{22} nanoclusters upon the 400 nm excitation. TA kinetic traces selected at specific probe wavelengths of (c) Au_{21}, (d) Au_{22} nanoclusters pumped at 400 nm and (e) Au_{21}, (f) Au_{22} nanoclusters pumped at 530 nm.
10. Crystal data and structure refinement for the $[Au_{21}(AdmS)_{12}(PPh_2py)_3]^+$ nanocluster.
11. Crystal data and structure refinement for the $[Au_{22}(AdmS)_{12}(PPh_2py)_4]^{2+}$ nanocluster.

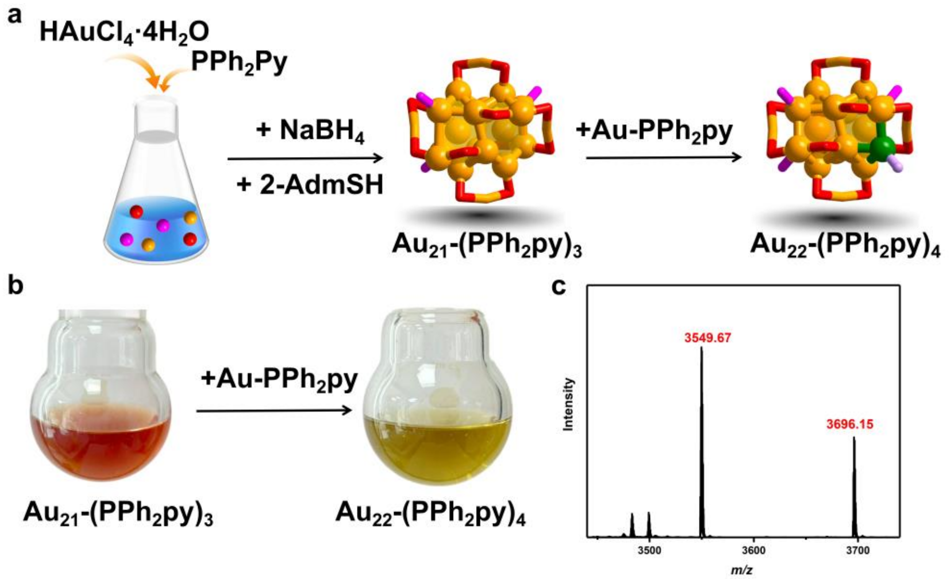

Supplementary Figure 1. (**a**) Synthesis of $[Au_{21}(AdmS)_{12}(PPh_2py)_3]^+$ and $[Au_{22}(AdmS)_{12}(PPh_2py)_4]^{2+}$ nanoclusters. (**b**) Upon the addition of the $AuPPh_2pyCl$ complex to Au_{21}, the solution color altered from reddish-brown to yellowish-green, indicating the cluster transformation from Au_{21} to Au_{22}. (**c**) ESI-MS results of the intermediate of the transformation from Au_{21} to Au_{22}.

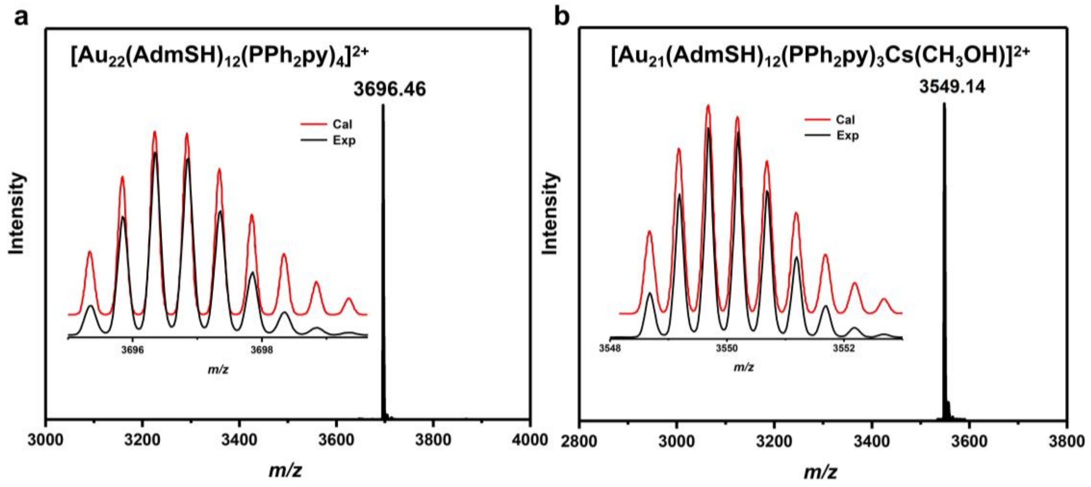

Supplementary Figure 2. Positive-ion mode ESI-MS results of (**a**) Au_{21} and (**b**) Au_{22} nanoclusters. Insets: the experimental (black trace) and simulated (red trace) isotopic patterns. For preparing the ESI samples, nanoclusters were dissolved in CH_2Cl_2 (1 mg mL^{-1}) and diluted (*v*/*v* = 1:1) with CH_3OH. The CH_3OH in the mass signal of the Au_{21} nanocluster should come from the solvent.

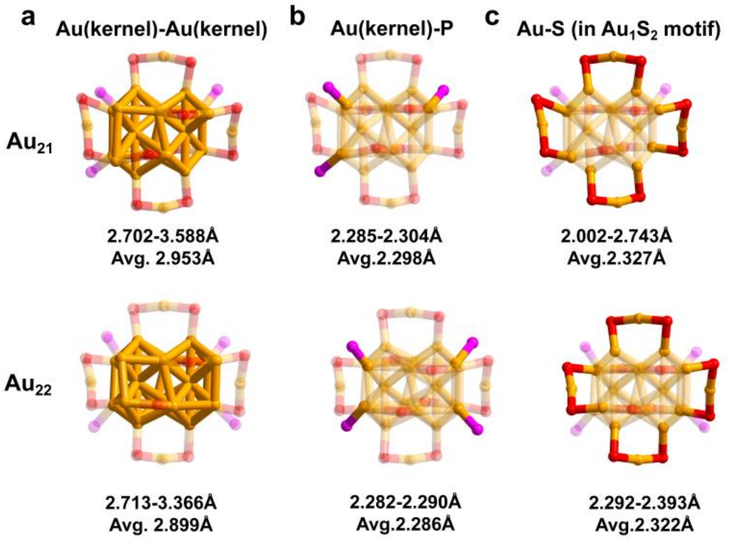

Supplementary Figure 3. Comparison of the lengths of (**a**) Au(kernel)-Au(kernel surface), (**b**) Au(kernel)-P, (**c**) Au-S (in Au_1S_2 motif) bonds in Au_{21} and Au_{22} nanoclusters. Color labels: light orange = Au; pink = P; red = S. all C, N and H atoms are omitted.

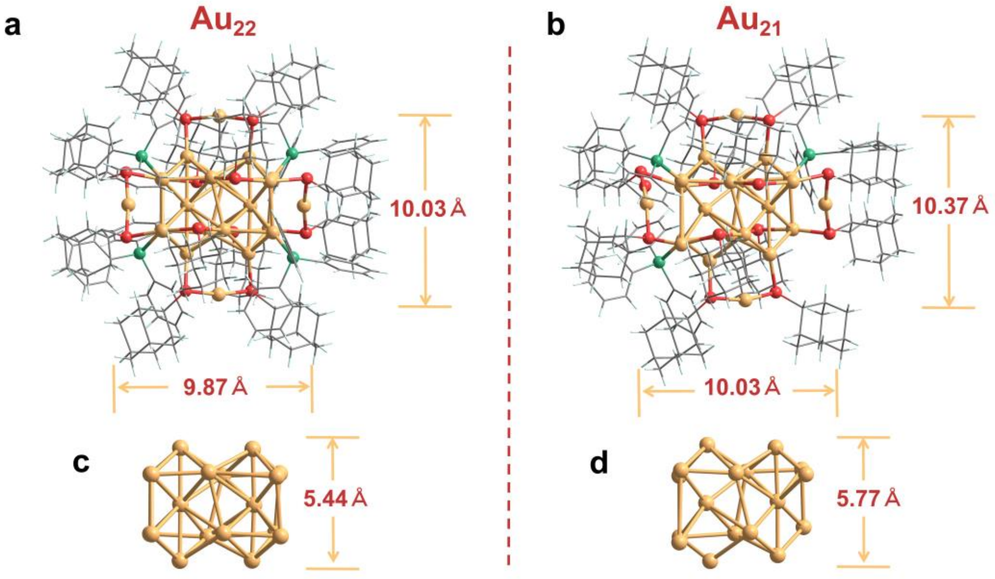

Supplementary Figure 4. Comparison between overall structures of the two nanoclusters. (**a**) The length of the Au_{22} nanocluster is 9.87 Å, and the width is 10.03 Å. (**b**) The length of the Au_{22} nanocluster is 10.03 Å, and the width is 10.37 Å. (**c**) The width of the Au_{22} core is 5.44 Å. (**d**) The width of the Au_{21} core is 5.77 Å.

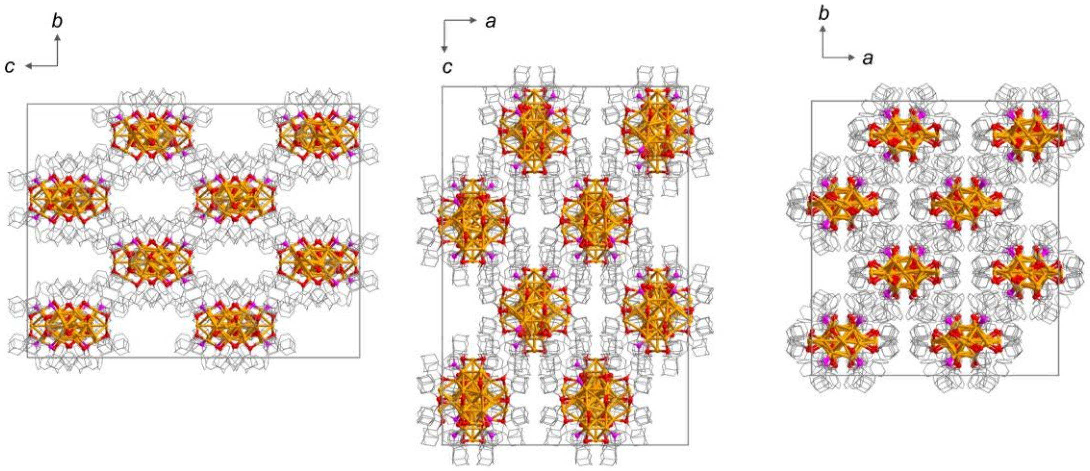

Supplementary Figure 5. Packing of $[Au_{21}(AdmS)_{12}(PPh_2py)_3]^+$ nanocluster molecules in the crystal lattice from the *a*-axis, *b*-axis and *c*-axis. Color labels: light orange = Au; pink = P; red = S; light grey sphere, N; grey sphere, C; white sphere, H.

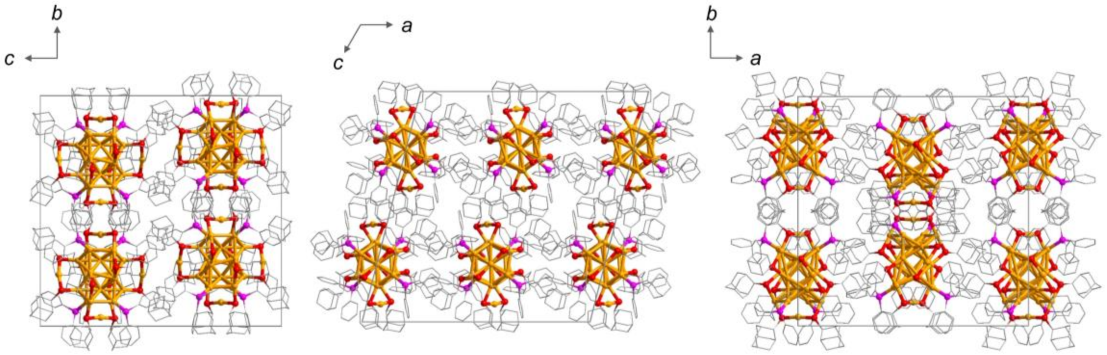

Supplementary Figure 6. Packing of $[Au_{22}(AdmS)_{12}(PPh_2py)_4]^{2+}$ nanocluster molecules in the crystal lattice from the *a*-axis, *b*-axis and *c*-axis. Color labels: light orange = Au; pink = P; red = S; light grey sphere, N; grey sphere, C; white sphere, H.

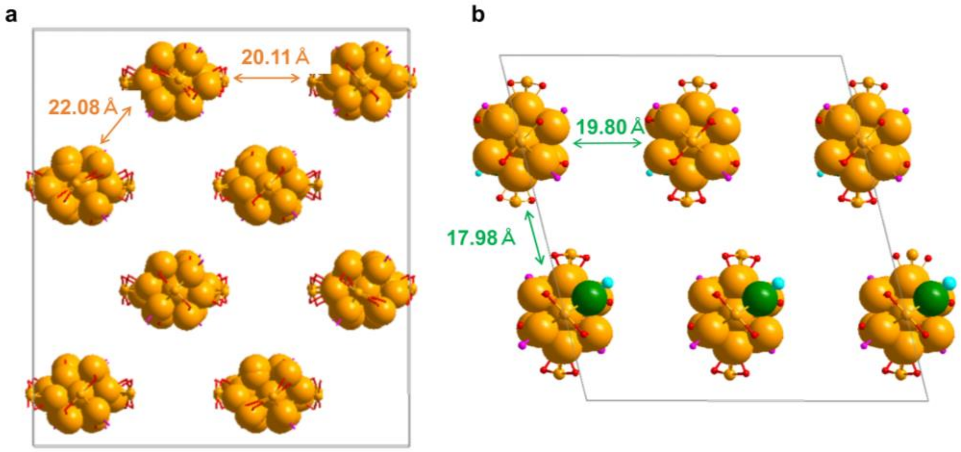

Supplementary Figure 7. Molecular spacing in the crystal lattice of (**a**) Au_{21} and (**b**) Au_{22} nanoclusters.

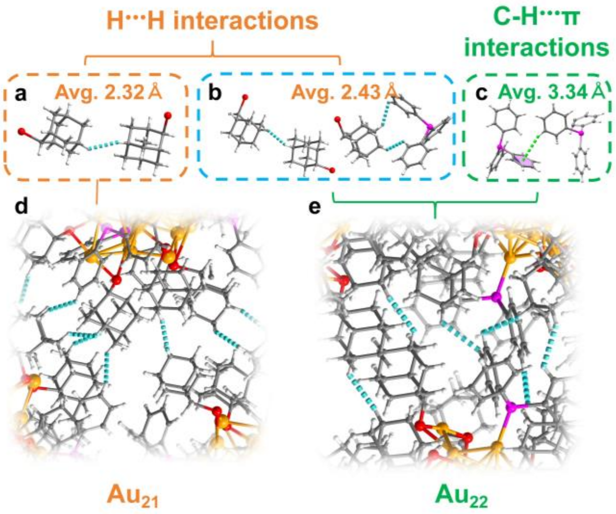

Supplementary Figure 8. Illustration of the intercluster interactions in Au_{21} and Au_{22} nanoclusters. The H···H interactions for (**a**) Au_{21} and (**b**) Au_{22} nanoclusters. (**c**) The C−H···π interactions for the Au_{22} nanocluster. The interaction of adjacent clusters in the crystal lattice of (**d**) Au_{21} and (**e**) Au_{22} nanoclusters.

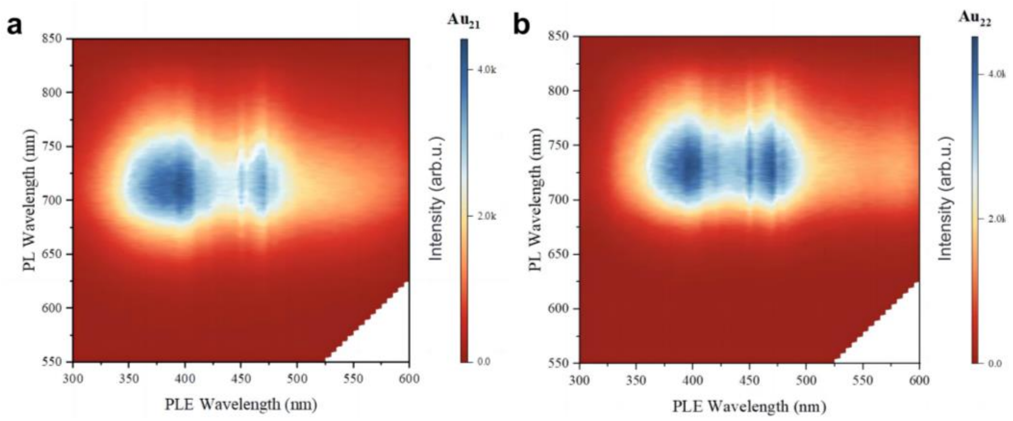

Supplementary Figure 9. Comparison of the excitation spectra (the excitation profiles obtained from the excitation-dependent emission spectra) of (**a**) Au_{21} and (**b**) Au_{22}.

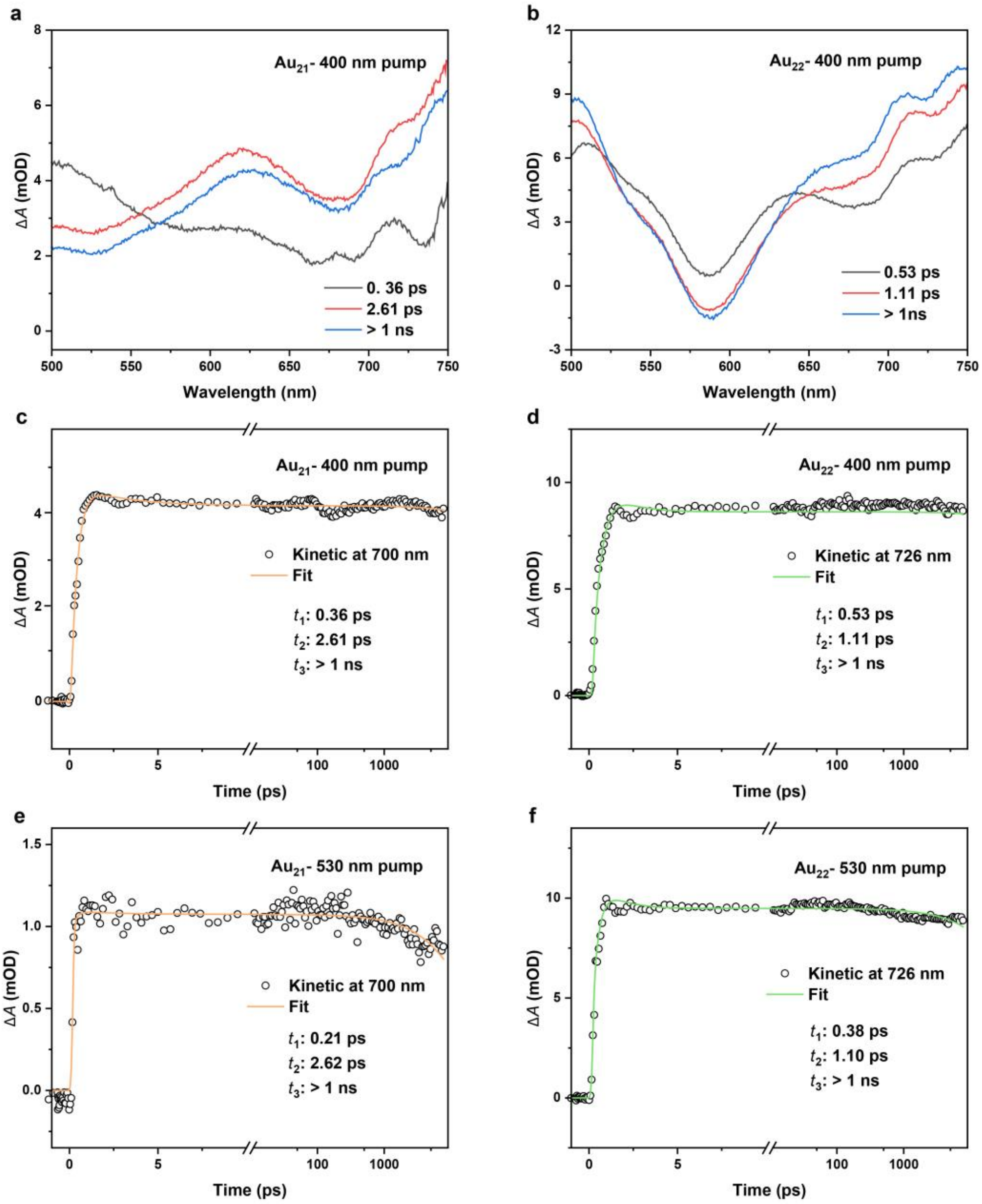

Supplementary Figure 10. Decay-associated spectra (DAS) obtained from global fitting of (**a**) Au_{21} and (**b**) Au_{22} nanoclusters upon the 400 nm excitation. TA kinetic traces selected at specific probe wavelengths of (**c**) Au_{21}, (**d**) Au_{22} nanoclusters pumped at 400 nm and (**e**) Au_{21}, (**f**) Au_{22} nanoclusters pumped at 530 nm.

Supplementary Table 1. Crystal data and structure refinement for the $[Au_{21}(AdmS)_{12}(PPh_2py)_3]^+$ nanocluster. The CCDC number of $[Au_{21}(AdmS)_{12}(PPh_2py)_3]^+$ is 2379695.

Crystal system	orthorhombic
Space group	*Fddd*
a/Å	38.946(8)
b/Å	43.238(9)
c/Å	56.684(11)
α/°	90
β/°	90
γ/°	90
Volume/Å^3	95454(33)
Z	16
P_{calc}g cm^{-3}	1.974
μ/mm^{-1}	25.408
F(000)	51760.0
Crystal size/mm^3	0.12× 0.08 × 0.1
Radiation	CuKα (λ = 1.54178)
Index ranges	$-31 \le h \le 46$, $-52 \le k \le 39$, $-67 \le l \le 60$
Final R indexes [I>=2σ (I)]	R_1 = 0.0749, wR_2 = 0.1946
Final R indexes [all data]	R_1 = 0.1032, wR_2 = 0.2117

For the crystal data of Au_{21}, there is an Au-PPh_2py unit following the positional disorder, located in Position 1 or 2 randomly, and the probability of each position is 50%. In this context, there are only three phosphine ligands in the crystal data of Au_{21}, which was further confirmed by ESI-MS measurements (Supplementary Figure 2).

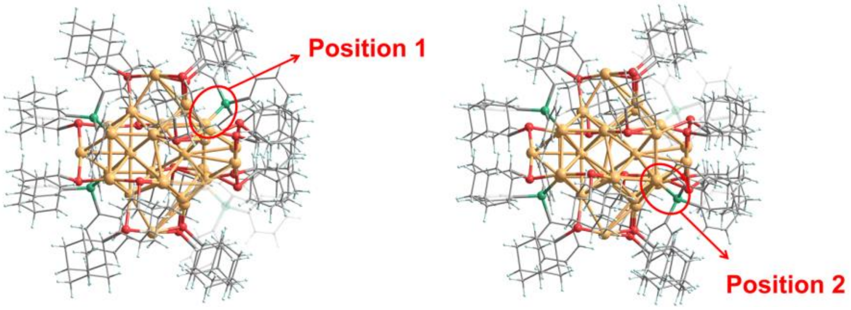

Supplementary Table 2. Crystal data and structure refinement for the $[Au_{22}(AdmS)_{12}(PPh_2py)_4]^{2+}$ nanocluster. The CCDC number of $[Au_{22}(AdmS)_{12}(PPh_2py)_4]^{2+}$ is 2379692.

Crystal system	monoclinic
Space group	*C2/c*
a/Å	28.529(7)
b/Å	27.479(15)
c/Å	29.712(12)
α/°	90
β/°	104.28(3)
γ/°	90
Volume/Å^3	22573(16)
Z	4
P_{calc}g cm^{-3}	2.175
μ/mm^{-1}	27.727
F(000)	13520.0
Crystal size/mm^3	0.5 × 0.5 × 0.5
Radiation	CuKα (λ = 1.54186)
Index ranges	-25 ≤ h ≤ 32, -31 ≤ k ≤ 29, -29 ≤ l ≤ 34
Final R indexes [I>=2σ (I)]	R_1 = 0.0392, wR_2 = 0.0926
Final R indexes [all data]	R_1 = 0.0554, wR_2 = 0.0961
