# Supplementary material for: Regulation of the photophysical dynamics of metal nanoclusters by manipulating single-point defects
Source: Nat Commun. 2025 Nov 17;16:10065. doi: 10.1038/s41467-025-65024-3 (PMC12623803; doi:10.1038/s41467-025-65024-3)
Supplement: Supplementary file 2 — Description of Additional Supplementary Files [file 41467_2025_65024_MOESM2_ESM.pdf]

Description of Additional Supplementary Files

File Name: Supplementary Data 1

Description: Crystallographic data for structures reported in this article, including Au21 and Au22 nanoclusters.
