# Supplementary material for: Regulation of the photophysical dynamics of metal nanoclusters by manipulating single-point defects
Source: Nat Commun. 2025 Nov 17;16:10065. doi: 10.1038/s41467-025-65024-3 (PMC12623803; doi:10.1038/s41467-025-65024-3)

checkCIF/PLATON report

You have not supplied any structure factors. As a result the full set of tests cannot be run.

THIS REPORT IS FOR GUIDANCE ONLY. IF USED AS PART OF A REVIEW PROCEDURE FOR PUBLICATION, IT SHOULD NOT REPLACE THE EXPERTISE OF AN EXPERIENCED CRYSTALLOGRAPHIC REFEREE.

No syntax errors found. CIF dictionary Interpreting this report

Datablock: aa_sq

```
Bond precision:   C-C = 0.0206 A          Wavelength=1.54178

Cell:               a=38.946(8)      b=43.238(9)      c=56.684(11)
                    alpha=90         beta=90          gamma=90
Temperature:        120 K
```

	Calculated	Reported
Volume	95453(34)	95454(33)
Space group	F d d d	F d d d
Hall group	-F 2uv 2vw	-F 2uv 2vw
Moiety formula	C171 H222 Au21 N3 P3 S12, 2(Br) [+ solvent]	C171 H222 Au21 N3 P3 S12, 2(Br1)
Sum formula	C171 H222 Au21 Br2 N3 P3 S12 [+ solvent]	C171 H222 Au21 Br2 N3 P3 S12
Mr	7093.30	7093.25
Dx,g cm-3	1.974	1.974
Z	16	16
Mu (mm-1)	25.408	25.408
F000	51760.0	51760.0
F000'	50434.73	
h,k,lmax	47,52,68	46,52,67
Nref	22476	22077
Tmin,Tmax	0.131,0.776	1.000,1.000
Tmin'	0.032	

```
Correction method= # Reported T Limits: Tmin=1.000 Tmax=1.000
AbsCorr = MULTI-SCAN

Data completeness= 0.982            Theta(max)= 69.573
```

R(reflections)= 0.0749(14678) wR2(reflections)= 0.2117(22077)

S = 0.970 Npar= 1222

The following ALERTS were generated. Each ALERT has the format
test-name_ALERT_alert-type_alert-level.
Click on the hyperlinks for more details of the test.

Alert level A

PLAT307_ALERT_2_A Isolated Metal Atom found in Structure (Unusual) Au3A Check

Author Response: Due to disorder, Au3A indeed coordinated with ligands, not isolated metal Atom.

PLAT308_ALERT_2_A Single Bonded Metal Atom in Structure (Unusual) Au4A Check

Author Response: Due to disorder, Au3A indeed coordinated with ligands, not single bonded Metal.

Alert level B

PLAT342_ALERT_3_B Low Bond Precision on C-C Bonds .... 0.02062 Ang.

Author Response: Bond precision might be affected by the residual between observed and calculated intensities, though empirical absorption correction were applied, the observed intensities would still be affected by the strong absorbers.

Alert level C

```
RINTA01_ALERT_3_C  The value of Rint is greater than 0.12
            Rint given   0.134
PLAT020_ALERT_3_C The Value of Rint is Greater Than 0.12 .........      0.134 Report
PLAT094_ALERT_2_C Ratio of Maximum / Minimum Residual Density ....       2.13 Report
PLAT215_ALERT_3_C Disordered Au2       has ADP max/min Ratio .....        3.1 Note
PLAT410_ALERT_2_C Short Intra H...H Contact  H7_7      ..H2_5      .     1.98 Ang.
                                              7/4-x,7/4-y,z  =     14_665 Check
```

Alert level G

```
PLAT002_ALERT_2_G Number of Distance or Angle Restraints on AtSite        80 Note
PLAT003_ALERT_2_G Number of Uiso or Uij Restrained non-H Atoms ...       126 Report
PLAT042_ALERT_1_G Calc. and Reported MoietyFormula Strings  Differ     Please Check
              Calc: C171 H222 Au21 N3 P3 S12, 2(Br)
              Rep.: C171 H222 Au21 N3 P3 S12, 2(Br1)
PLAT072_ALERT_2_G SHELXL First  Parameter in WGHT  Unusually Large       0.14 Report
```

```
PLAT171_ALERT_4_G The CIF-Embedded .res File Contains EADP Records          2 Report
PLAT172_ALERT_4_G The CIF-Embedded .res File Contains DFIX Records          1 Report
PLAT173_ALERT_4_G The CIF-Embedded .res File Contains DANG Records         14 Report
PLAT178_ALERT_4_G The CIF-Embedded .res File Contains SIMU Records          2 Report
PLAT300_ALERT_4_G Atom Site Occupancy of Au1         Constrained at        0.5 Check
PLAT300_ALERT_4_G Atom Site Occupancy of Au2         Constrained at        0.5 Check
PLAT300_ALERT_4_G Atom Site Occupancy of S1_1        Constrained at        0.5 Check
PLAT300_ALERT_4_G Atom Site Occupancy of S1_2        Constrained at        0.5 Check
PLAT300_ALERT_4_G Atom Site Occupancy of S1_10       Constrained at        0.5 Check
PLAT300_ALERT_4_G Atom Site Occupancy of S1_11       Constrained at        0.5 Check
PLAT300_ALERT_4_G Atom Site Occupancy of P1_8        Constrained at        0.5 Check
PLAT300_ALERT_4_G Atom Site Occupancy of N1_8        Constrained at        0.5 Check
PLAT300_ALERT_4_G Atom Site Occupancy of C2_8        Constrained at        0.5 Check
PLAT300_ALERT_4_G Atom Site Occupancy of C1_8        Constrained at        0.5 Check
PLAT300_ALERT_4_G Atom Site Occupancy of C5_8        Constrained at        0.5 Check
PLAT300_ALERT_4_G Atom Site Occupancy of C4_8        Constrained at        0.5 Check
PLAT300_ALERT_4_G Atom Site Occupancy of C3_8        Constrained at        0.5 Check
PLAT300_ALERT_4_G Atom Site Occupancy of C7_8        Constrained at        0.5 Check
PLAT300_ALERT_4_G Atom Site Occupancy of C6_8        Constrained at        0.5 Check
PLAT300_ALERT_4_G Atom Site Occupancy of C11_8       Constrained at        0.5 Check
PLAT300_ALERT_4_G Atom Site Occupancy of C10_8       Constrained at        0.5 Check
PLAT300_ALERT_4_G Atom Site Occupancy of C9_8        Constrained at        0.5 Check
PLAT300_ALERT_4_G Atom Site Occupancy of C8_8        Constrained at        0.5 Check
PLAT300_ALERT_4_G Atom Site Occupancy of C15_8       Constrained at        0.5 Check
PLAT300_ALERT_4_G Atom Site Occupancy of C16_8       Constrained at        0.5 Check
PLAT300_ALERT_4_G Atom Site Occupancy of C17_8       Constrained at        0.5 Check
PLAT300_ALERT_4_G Atom Site Occupancy of C12_8       Constrained at        0.5 Check
PLAT300_ALERT_4_G Atom Site Occupancy of C13_8       Constrained at        0.5 Check
PLAT300_ALERT_4_G Atom Site Occupancy of C14_8       Constrained at        0.5 Check
PLAT300_ALERT_4_G Atom Site Occupancy of C1_1        Constrained at        0.5 Check
PLAT300_ALERT_4_G Atom Site Occupancy of C2_1        Constrained at        0.5 Check
PLAT300_ALERT_4_G Atom Site Occupancy of C3_1        Constrained at        0.5 Check
PLAT300_ALERT_4_G Atom Site Occupancy of C4_1        Constrained at        0.5 Check
PLAT300_ALERT_4_G Atom Site Occupancy of C5_1        Constrained at        0.5 Check
PLAT300_ALERT_4_G Atom Site Occupancy of C6_1        Constrained at        0.5 Check
PLAT300_ALERT_4_G Atom Site Occupancy of C7_1        Constrained at        0.5 Check
PLAT300_ALERT_4_G Atom Site Occupancy of C8_1        Constrained at        0.5 Check
PLAT300_ALERT_4_G Atom Site Occupancy of C9_1        Constrained at        0.5 Check
PLAT300_ALERT_4_G Atom Site Occupancy of C10_1       Constrained at        0.5 Check
PLAT300_ALERT_4_G Atom Site Occupancy of C1_2        Constrained at        0.5 Check
PLAT300_ALERT_4_G Atom Site Occupancy of C2_2        Constrained at        0.5 Check
PLAT300_ALERT_4_G Atom Site Occupancy of C3_2        Constrained at        0.5 Check
PLAT300_ALERT_4_G Atom Site Occupancy of C4_2        Constrained at        0.5 Check
PLAT300_ALERT_4_G Atom Site Occupancy of C5_2        Constrained at        0.5 Check
PLAT300_ALERT_4_G Atom Site Occupancy of C6_2        Constrained at        0.5 Check
PLAT300_ALERT_4_G Atom Site Occupancy of C7_2        Constrained at        0.5 Check
PLAT300_ALERT_4_G Atom Site Occupancy of C8_2        Constrained at        0.5 Check
PLAT300_ALERT_4_G Atom Site Occupancy of C9_2        Constrained at        0.5 Check
PLAT300_ALERT_4_G Atom Site Occupancy of C10_2       Constrained at        0.5 Check
PLAT300_ALERT_4_G Atom Site Occupancy of C1_10       Constrained at        0.5 Check
PLAT300_ALERT_4_G Atom Site Occupancy of C2_10       Constrained at        0.5 Check
PLAT300_ALERT_4_G Atom Site Occupancy of C3_10       Constrained at        0.5 Check
PLAT300_ALERT_4_G Atom Site Occupancy of C4_10       Constrained at        0.5 Check
PLAT300_ALERT_4_G Atom Site Occupancy of C5_10       Constrained at        0.5 Check
PLAT300_ALERT_4_G Atom Site Occupancy of C6_10       Constrained at        0.5 Check
PLAT300_ALERT_4_G Atom Site Occupancy of C7_10       Constrained at        0.5 Check
PLAT300_ALERT_4_G Atom Site Occupancy of C8_10       Constrained at        0.5 Check
```

```
PLAT300_ALERT_4_G Atom Site Occupancy of C9_10      Constrained at        0.5 Check
PLAT300_ALERT_4_G Atom Site Occupancy of C10_10     Constrained at        0.5 Check
PLAT300_ALERT_4_G Atom Site Occupancy of C1_11      Constrained at        0.5 Check
PLAT300_ALERT_4_G Atom Site Occupancy of C2_11      Constrained at        0.5 Check
PLAT300_ALERT_4_G Atom Site Occupancy of C3_11      Constrained at        0.5 Check
PLAT300_ALERT_4_G Atom Site Occupancy of C4_11      Constrained at        0.5 Check
PLAT300_ALERT_4_G Atom Site Occupancy of C5_11      Constrained at        0.5 Check
PLAT300_ALERT_4_G Atom Site Occupancy of C6_11      Constrained at        0.5 Check
PLAT300_ALERT_4_G Atom Site Occupancy of C7_11      Constrained at        0.5 Check
PLAT300_ALERT_4_G Atom Site Occupancy of C8_11      Constrained at        0.5 Check
PLAT300_ALERT_4_G Atom Site Occupancy of C9_11      Constrained at        0.5 Check
PLAT300_ALERT_4_G Atom Site Occupancy of C10_11     Constrained at        0.5 Check
PLAT300_ALERT_4_G Atom Site Occupancy of H2_8       Constrained at        0.5 Check
PLAT300_ALERT_4_G Atom Site Occupancy of H5_8       Constrained at        0.5 Check
PLAT300_ALERT_4_G Atom Site Occupancy of H4_8       Constrained at        0.5 Check
PLAT300_ALERT_4_G Atom Site Occupancy of H3_8       Constrained at        0.5 Check
PLAT300_ALERT_4_G Atom Site Occupancy of H7_8       Constrained at        0.5 Check
PLAT300_ALERT_4_G Atom Site Occupancy of H11_8      Constrained at        0.5 Check
PLAT300_ALERT_4_G Atom Site Occupancy of H10_8      Constrained at        0.5 Check
PLAT300_ALERT_4_G Atom Site Occupancy of H9_8       Constrained at        0.5 Check
PLAT300_ALERT_4_G Atom Site Occupancy of H8_8       Constrained at        0.5 Check
PLAT300_ALERT_4_G Atom Site Occupancy of H15_8      Constrained at        0.5 Check
PLAT300_ALERT_4_G Atom Site Occupancy of H16_8      Constrained at        0.5 Check
PLAT300_ALERT_4_G Atom Site Occupancy of H17_8      Constrained at        0.5 Check
PLAT300_ALERT_4_G Atom Site Occupancy of H13_8      Constrained at        0.5 Check
PLAT300_ALERT_4_G Atom Site Occupancy of H14_8      Constrained at        0.5 Check
PLAT300_ALERT_4_G Atom Site Occupancy of H1_1       Constrained at        0.5 Check
PLAT300_ALERT_4_G Atom Site Occupancy of H2_1       Constrained at        0.5 Check
PLAT300_ALERT_4_G Atom Site Occupancy of H3A_1      Constrained at        0.5 Check
PLAT300_ALERT_4_G Atom Site Occupancy of H3B_1      Constrained at        0.5 Check
PLAT300_ALERT_4_G Atom Site Occupancy of H4_1       Constrained at        0.5 Check
PLAT300_ALERT_4_G Atom Site Occupancy of H5A_1      Constrained at        0.5 Check
PLAT300_ALERT_4_G Atom Site Occupancy of H5B_1      Constrained at        0.5 Check
PLAT300_ALERT_4_G Atom Site Occupancy of H6_1       Constrained at        0.5 Check
PLAT300_ALERT_4_G Atom Site Occupancy of H7A_1      Constrained at        0.5 Check
PLAT300_ALERT_4_G Atom Site Occupancy of H7B_1      Constrained at        0.5 Check
PLAT300_ALERT_4_G Atom Site Occupancy of H8_1       Constrained at        0.5 Check
PLAT300_ALERT_4_G Atom Site Occupancy of H9A_1      Constrained at        0.5 Check
PLAT300_ALERT_4_G Atom Site Occupancy of H9B_1      Constrained at        0.5 Check
PLAT300_ALERT_4_G Atom Site Occupancy of H10A_1     Constrained at        0.5 Check
PLAT300_ALERT_4_G Atom Site Occupancy of H10B_1     Constrained at        0.5 Check
PLAT300_ALERT_4_G Atom Site Occupancy of H1_2       Constrained at        0.5 Check
PLAT300_ALERT_4_G Atom Site Occupancy of H2_2       Constrained at        0.5 Check
PLAT300_ALERT_4_G Atom Site Occupancy of H3A_2      Constrained at        0.5 Check
PLAT300_ALERT_4_G Atom Site Occupancy of H3B_2      Constrained at        0.5 Check
PLAT300_ALERT_4_G Atom Site Occupancy of H4_2       Constrained at        0.5 Check
PLAT300_ALERT_4_G Atom Site Occupancy of H5A_2      Constrained at        0.5 Check
PLAT300_ALERT_4_G Atom Site Occupancy of H5B_2      Constrained at        0.5 Check
PLAT300_ALERT_4_G Atom Site Occupancy of H6_2       Constrained at        0.5 Check
PLAT300_ALERT_4_G Atom Site Occupancy of H7A_2      Constrained at        0.5 Check
PLAT300_ALERT_4_G Atom Site Occupancy of H7B_2      Constrained at        0.5 Check
PLAT300_ALERT_4_G Atom Site Occupancy of H8_2       Constrained at        0.5 Check
PLAT300_ALERT_4_G Atom Site Occupancy of H9A_2      Constrained at        0.5 Check
PLAT300_ALERT_4_G Atom Site Occupancy of H9B_2      Constrained at        0.5 Check
PLAT300_ALERT_4_G Atom Site Occupancy of H10A_2     Constrained at        0.5 Check
PLAT300_ALERT_4_G Atom Site Occupancy of H10B_2     Constrained at        0.5 Check
PLAT300_ALERT_4_G Atom Site Occupancy of H1_10      Constrained at        0.5 Check
```

```
PLAT300_ALERT_4_G Atom Site Occupancy of H2_10      Constrained at        0.5 Check
PLAT300_ALERT_4_G Atom Site Occupancy of H3A_10     Constrained at        0.5 Check
PLAT300_ALERT_4_G Atom Site Occupancy of H3B_10     Constrained at        0.5 Check
PLAT300_ALERT_4_G Atom Site Occupancy of H4_10      Constrained at        0.5 Check
PLAT300_ALERT_4_G Atom Site Occupancy of H5A_10     Constrained at        0.5 Check
PLAT300_ALERT_4_G Atom Site Occupancy of H5B_10     Constrained at        0.5 Check
PLAT300_ALERT_4_G Atom Site Occupancy of H6_10      Constrained at        0.5 Check
PLAT300_ALERT_4_G Atom Site Occupancy of H7A_10     Constrained at        0.5 Check
PLAT300_ALERT_4_G Atom Site Occupancy of H7B_10     Constrained at        0.5 Check
PLAT300_ALERT_4_G Atom Site Occupancy of H8_10      Constrained at        0.5 Check
PLAT300_ALERT_4_G Atom Site Occupancy of H9A_10     Constrained at        0.5 Check
PLAT300_ALERT_4_G Atom Site Occupancy of H9B_10     Constrained at        0.5 Check
PLAT300_ALERT_4_G Atom Site Occupancy of H10A_10    Constrained at        0.5 Check
PLAT300_ALERT_4_G Atom Site Occupancy of H10B_10    Constrained at        0.5 Check
PLAT300_ALERT_4_G Atom Site Occupancy of H1_11      Constrained at        0.5 Check
PLAT300_ALERT_4_G Atom Site Occupancy of H2_11      Constrained at        0.5 Check
PLAT300_ALERT_4_G Atom Site Occupancy of H3A_11     Constrained at        0.5 Check
PLAT300_ALERT_4_G Atom Site Occupancy of H3B_11     Constrained at        0.5 Check
PLAT300_ALERT_4_G Atom Site Occupancy of H4_11      Constrained at        0.5 Check
PLAT300_ALERT_4_G Atom Site Occupancy of H5A_11     Constrained at        0.5 Check
PLAT300_ALERT_4_G Atom Site Occupancy of H5B_11     Constrained at        0.5 Check
PLAT300_ALERT_4_G Atom Site Occupancy of H6_11      Constrained at        0.5 Check
PLAT300_ALERT_4_G Atom Site Occupancy of H7A_11     Constrained at        0.5 Check
PLAT300_ALERT_4_G Atom Site Occupancy of H7B_11     Constrained at        0.5 Check
PLAT300_ALERT_4_G Atom Site Occupancy of H8_11      Constrained at        0.5 Check
PLAT300_ALERT_4_G Atom Site Occupancy of H9A_11     Constrained at        0.5 Check
PLAT300_ALERT_4_G Atom Site Occupancy of H9B_11     Constrained at        0.5 Check
PLAT300_ALERT_4_G Atom Site Occupancy of H10A_11    Constrained at        0.5 Check
PLAT300_ALERT_4_G Atom Site Occupancy of H10B_11    Constrained at        0.5 Check
PLAT301_ALERT_3_G Main Residue  Disorder .............(Resd    1)        34% Note
PLAT302_ALERT_4_G Anion/Solvent/Minor-Residue Disorder (Resd    3)       100% Note
PLAT302_ALERT_4_G Anion/Solvent/Minor-Residue Disorder (Resd    4)       100% Note
PLAT304_ALERT_4_G Non-Integer Number of Atoms in ..... (Resd    2)       0.50 Check
PLAT304_ALERT_4_G Non-Integer Number of Atoms in ..... (Resd    3)       0.33 Check
PLAT304_ALERT_4_G Non-Integer Number of Atoms in ..... (Resd    4)       0.17 Check
PLAT606_ALERT_4_G Solvent Accessible VOID(S) in Structure ........          ! Info
PLAT764_ALERT_4_G Overcomplete CIF Bond List Detected (Rep/Expd) .       1.19 Ratio
PLAT779_ALERT_4_G Suspect or Irrelevant (Bond) Angle(s) in CIF ...      18.65 Deg.
              AU1  -S1_1 -AU1    14_665   1_555   1_555 ......     #  545 Check
PLAT779_ALERT_4_G Suspect or Irrelevant (Bond) Angle(s) in CIF ...      26.84 Deg.
              AU1  -S1_10-AU1    14_665   1_555   1_555 ......     #  930 Check
PLAT794_ALERT_5_G Tentative Bond Valency for Au11      (III)     .       2.63 Info
PLAT811_ALERT_5_G No ADDSYM Analysis: Too Many Excluded Atoms ....          ! Info
PLAT860_ALERT_3_G Number of Least-Squares Restraints ............        1084 Note
PLAT869_ALERT_4_G ALERTS Related to the Use of SQUEEZE  Suppressed          ! Info
PLAT883_ALERT_1_G No Info/Value for _atom_sites_solution_primary .     Please Do !
PLAT933_ALERT_2_G Number of HKL-OMIT Records in Embedded .res File          9 Note
                6 18  8,   7 21  3,   8  0  0,  15  1  1,  17  3  5,   6  8 12,
                0  4  8,   0  2 10,   2  0 14,
```

---

2 **ALERT level A** = Most likely a serious problem - resolve or explain
1 **ALERT level B** = A potentially serious problem, consider carefully
5 **ALERT level C** = Check. Ensure it is not caused by an omission or oversight
163 **ALERT level G** = General information/check it is not something unexpected

2 ALERT type 1 CIF construction/syntax error, inconsistent or missing data

```
  8 ALERT type 2 Indicator that the structure model may be wrong or deficient
  6 ALERT type 3 Indicator that the structure quality may be low
153 ALERT type 4 Improvement, methodology, query or suggestion
  2 ALERT type 5 Informative message, check
```

It is advisable to attempt to resolve as many as possible of the alerts in all categories. Often the minor alerts point to easily fixed oversights, errors and omissions in your CIF or refinement strategy, so attention to these fine details can be worthwhile. In order to resolve some of the more serious problems it may be necessary to carry out additional measurements or structure refinements. However, the purpose of your study may justify the reported deviations and the more serious of these should normally be commented upon in the discussion or experimental section of a paper or in the "special_details" fields of the CIF. checkCIF was carefully designed to identify outliers and unusual parameters, but every test has its limitations and alerts that are not important in a particular case may appear. Conversely, the absence of alerts does not guarantee there are no aspects of the results needing attention. It is up to the individual to critically assess their own results and, if necessary, seek expert advice.

Publication of your CIF in IUCr journals

A basic structural check has been run on your CIF. These basic checks will be run on all CIFs submitted for publication in IUCr journals (*Acta Crystallographica*, *Journal of Applied Crystallography*, *Journal of Synchrotron Radiation*); however, if you intend to submit to *Acta Crystallographica Section C* or *E* or *IUCrData*, you should make sure that full publication checks are run on the final version of your CIF prior to submission.

Publication of your CIF in other journals

Please refer to the *Notes for Authors* of the relevant journal for any special instructions relating to CIF submission.

PLATON version of 06/01/2024; check.def file version of 05/01/2024

Datablock aa_sq - ellipsoid plot

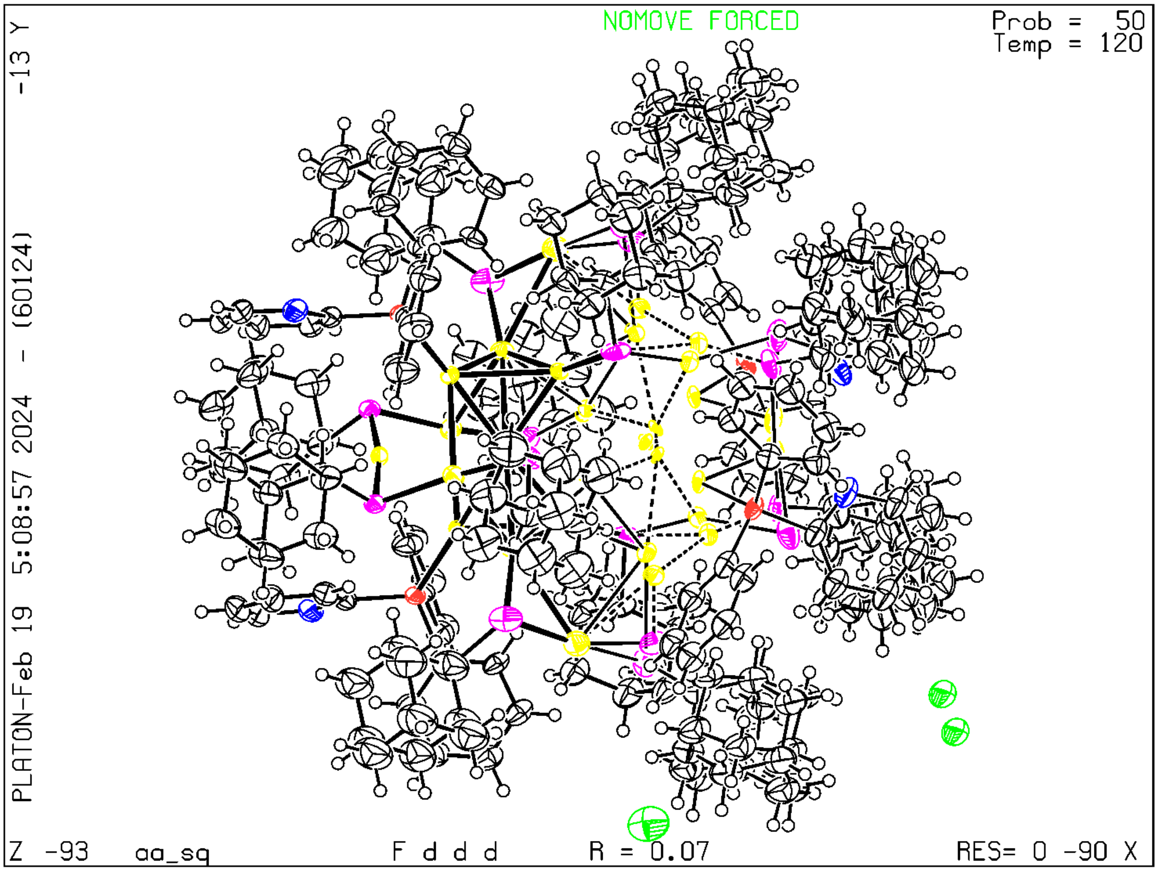

Supplement: Supplementary file 3 — Supplementary Data 1 [file 41467_2025_65024_MOESM3_ESM.zip › Supplementary Data 1/checkCIF Au21.pdf]
