# Supplementary material for: Regulation of the photophysical dynamics of metal nanoclusters by manipulating single-point defects
Source: Nat Commun. 2025 Nov 17;16:10065. doi: 10.1038/s41467-025-65024-3 (PMC12623803; doi:10.1038/s41467-025-65024-3)

checkCIF/PLATON report

Structure factors have been supplied for datablock(s) 1

THIS REPORT IS FOR GUIDANCE ONLY. IF USED AS PART OF A REVIEW PROCEDURE FOR PUBLICATION, IT SHOULD NOT REPLACE THE EXPERTISE OF AN EXPERIENCED CRYSTALLOGRAPHIC REFEREE.

No syntax errors found. CIF dictionary Interpreting this report

Datablock: 1

Bond precision:	C-C = 0.0187 A		Wavelength=1.54186
Cell:	a=28.529(7)	b=27.479(15)	c=29.712(12)
	alpha=90	beta=104.28(3)	gamma=90
Temperature:	120 K		

	Calculated	Reported
Volume	22573(17)	22573(16)
Space group	C 2/c	C 1 2/c 1
Hall group	-C 2yc	-C 2yc
Moiety formula	C188 H234 Au22 N4 P4 S12 [+ solvent]	C188 H234 Au22 N4 P4 S12
Sum formula	C188 H234 Au22 N4 P4 S12 [+ solvent]	C188 H234 Au22 N4 P4 S12
Mr	7391.75	7391.64
Dx,g cm-3	2.175	2.175
Z	4	4
Mu (mm-1)	27.727	27.727
F000	13520.0	13520.0
F000'	13179.09	
h,k,lmax	32,31,34	32,31,34
Nref	18013	17681
Tmin,Tmax	0.000,0.000	1.000,1.000
Tmin'	0.000	

Correction method= # Reported T Limits: Tmin=1.000 Tmax=1.000
AbsCorr = MULTI-SCAN

Data completeness= 0.982 Theta(max)= 62.497

R(reflections)= 0.0392(12223)

wR2(reflections)= 0.0961(17681)

S = 0.885 Npar= 1002

The following ALERTS were generated. Each ALERT has the format
test-name_ALERT_alert-type_alert-level.
Click on the hyperlinks for more details of the test.

Alert level B

```
PLAT910_ALERT_3_B Missing # of FCF Reflection(s) Below Theta(Min).         23 Note
                   0  2  0,   1  1  0,   1  3  0,   2  0  0,   2  2  0,   3  1  0,
                  -3  1  1,  -2  2  1,  -1  1  1,  -1  3  1,   0  2  1,   1  1  1,
                   1  3  1,   2  2  1,  -3  1  2,  -2  0  2,  -2  2  2,  -1  1  2,
                   0  0  2,   0  2  2,   1  1  2,   2  0  2,  -1  1  3,
```

Alert level C

```
THETM01_ALERT_3_C  The value of sine(theta_max)/wavelength is less than 0.590
            Calculated sin(theta_max)/wavelength =    0.5753
PLAT230_ALERT_2_C Hirshfeld Test Diff for    P00L     --C40      .        6.5 s.u.
PLAT230_ALERT_2_C Hirshfeld Test Diff for    P00L     --C63      .        6.5 s.u.
PLAT230_ALERT_2_C Hirshfeld Test Diff for    P00L     --C02N     .        6.8 s.u.
PLAT234_ALERT_4_C Large Hirshfeld Difference N02C     --C00Z     .       0.22 Ang.
PLAT234_ALERT_4_C Large Hirshfeld Difference C40      --C2AA     .       0.17 Ang.
PLAT234_ALERT_4_C Large Hirshfeld Difference C49      --C02N     .       0.16 Ang.
PLAT234_ALERT_4_C Large Hirshfeld Difference C02Q     --C01Y     .       0.19 Ang.
PLAT234_ALERT_4_C Large Hirshfeld Difference C63      --C01P     .       0.17 Ang.
PLAT241_ALERT_2_C High   'MainMol' Ueq as Compared to Neighbors of       N02C Check
PLAT242_ALERT_2_C Low    'MainMol' Ueq as Compared to Neighbors of       P00L Check
PLAT242_ALERT_2_C Low    'MainMol' Ueq as Compared to Neighbors of       C01Y Check
PLAT342_ALERT_3_C Low Bond Precision on  C-C Bonds ..............    0.01873 Ang.
PLAT361_ALERT_2_C Long   C(sp3)-C(sp3) Bond  C01W      - C02L     .       1.65 Ang.
PLAT911_ALERT_3_C Missing FCF Refl Between Thmin & STh/L=    0.575        309 Report
                   0  8  0,   1  7  0,   3  3  0,   4  4  0,  -2  4  1,   0  6  1,
                   3  1  1,   6  2  1,  -8  0  2,  -2  4  2,  -2  6  2,  -1  3  2,
                   2  2  2,   3  1  2,   3 13  2,   4 14  2, -13 11  3,  -7  1  3,
                  -3  1  3,  -3  5  3,  -2  2  3,   0  2  3,   0  6  3,   5  1  3,
                   5 15  3,   6 16  3, -24  2  4,  -4  0  4,  -2  2  4,  -1  1  4,
                   0  2  4,   3 15  4,   4 16  4,   5 17  4,  -6  4  5,  -6 18  5,
                  -3  1  5,   2  2  5,   5 17  5,   6 18  5, -26  0  6,  -7 17  6,
                  -6 18  6,  -3  1  6,  -1  1  6,   0  2  6,   1  3  6,   2  4  6,
                 -15  5  7,  -9  3  7,  -5  1  7,  -3  1  7,  -3 11  7,  -2 12  7,
                  -1  3  7, -11  3  8,  -5  1  8,  -4  0  8,  -4  2  8,  -4 12  8,
                  -3  1  8,  -3 13  8,  28  0  8,  29  1  8, -13  3  9,  -6  2  9,
                  -5  1  9,  -4  2  9,  -3  1  9,   5  5  9,   6  6  9, -15 13 10,
                  -5  1 10,  -4  0 10,   6  6 10,   7  7 10,   8  8 10,   9  9 10,
                  16  0 10, -15 13 11,   5  7 11,   6  8 11,   7  9 11,   8 10 11,
                  20  4 11,  25  7 11, -22  0 12, -12  4 12, -11  5 12,  -6  0 12,
                  -4  0 12,  -2  0 12,  -1 29 12,   8 10 12,  20  4 12,  24  6 12,
PLAT913_ALERT_3_C Missing # of Very Strong Reflections in FCF ....          4 Note
                   2  0  0,  -1  1  1,   1  1  1,   0  0  2,
PLAT934_ALERT_3_C Number of (Iobs-Icalc)/Sigma(W) > 10 Outliers ..          1 Check
                   5  1 15,
PLAT971_ALERT_2_C Check Calcd Resid. Dens.  1.84Ang From C40            1.73 eA-3
```

```
PLAT971_ALERT_2_C Check Calcd Resid. Dens.  0.98Ang From Au07           1.65 eA-3
PLAT971_ALERT_2_C Check Calcd Resid. Dens.  1.16Ang From C98            1.56 eA-3
PLAT972_ALERT_2_C Check Calcd Resid. Dens.  0.94Ang From Au03          -1.73 eA-3
PLAT977_ALERT_2_C Check Negative Difference Density on H1A      .      -0.31 eA-3
PLAT977_ALERT_2_C Check Negative Difference Density on H2       .      -0.53 eA-3
PLAT977_ALERT_2_C Check Negative Difference Density on H00A     .      -0.37 eA-3
PLAT977_ALERT_2_C Check Negative Difference Density on H013     .      -0.31 eA-3
PLAT977_ALERT_2_C Check Negative Difference Density on H014     .      -0.43 eA-3
PLAT977_ALERT_2_C Check Negative Difference Density on H01C     .      -0.33 eA-3
PLAT977_ALERT_2_C Check Negative Difference Density on H01K     .      -0.32 eA-3
```

Alert level G

```
PLAT003_ALERT_2_G Number of Uiso or Uij Restrained non-H Atoms ...         95 Report
PLAT177_ALERT_4_G The CIF-Embedded .res File Contains DELU Records          2 Report
PLAT178_ALERT_4_G The CIF-Embedded .res File Contains SIMU Records          3 Report
PLAT186_ALERT_4_G The CIF-Embedded .res File Contains ISOR Records          6 Report
PLAT188_ALERT_3_G A Non-default SIMU Restraint Value has been used     0.0100 Report
PLAT188_ALERT_3_G A Non-default SIMU Restraint Value has been used     0.0100 Report
PLAT188_ALERT_3_G A Non-default SIMU Restraint Value has been used     0.0100 Report
PLAT343_ALERT_2_G Unusual sp?     Angle Range in Main Residue for       C01P Check
PLAT606_ALERT_4_G Solvent Accessible VOID(S) in Structure ........         ! Info
PLAT720_ALERT_4_G Number of Unusual/Non-Standard Labels ..........       159 Note
              Au01    Au02    Au03    Au04    Au05    Au06    Au07    Au08
              Au09    Au0A    Au0B    Au0C    Au0D    S00E    S00F    S00G
              S00H    P00I    S00J    S00K    P00L    C00Q    H00Q    C00R
              H00R    C00S    H00S    C00T    C00U    H00A    H00B    C00V
              H00V    C00W    H00W    C00Y    H00Y    C00Z    C010    H010
              C011    H011    C013    H013    C014    H014    C015    H01A
              H01B    C017    H017    C018    C019    H01C    H01D    C01A
              H01E    H01F    C01C    H01G    C01D    H01H    H01I    C01F
              H01J    C01G    H01K    H01L    C01H    H01M    C01I    H01N
              C01J    H01O    H01P    C01K    H01Q    C01L    H01R    C01M
              H01S    H01T    C01N    H01U    C01O    H01V    C01Q    H01W
              C01R    H01X    H01Y    C01S    H01Z    C01T    H01     C01U
              C01V    C01W    C01X    Ha      C020    H02A    H02B    C021
              H02C    H02D    C022    H02E    H02F    C023    H023    C024
              H024    C026    H02G    H02H    C027    H027    C028    H028
              C029    H029    C02A    H02I    C02B    H02J    N02C    C02D
              H02K    C02E    H02L    C02G    H02M    C02H    H02N    C02I
              H02O    H02P    C02L    H02Q    H02R    C02M    H02S    C02Q
              H02T    C01Y    N016    C2AA    H2AA    C5AA    H5AA    H5AB
              C0AA    H0AA    H0AB    C1AA    H1AA    C02N    C01P
PLAT794_ALERT_5_G Tentative Bond Valency for Au07      (III)     .       2.64 Info
PLAT860_ALERT_3_G Number of Least-Squares Restraints .............      1203 Note
PLAT868_ALERT_4_G ALERTS Due to the Use of _smtbx_masks Suppressed          ! Info
PLAT909_ALERT_3_G Percentage of I>2sig(I) Data at Theta(Max) Still        56% Note
PLAT933_ALERT_2_G Number of HKL-OMIT Records in Embedded .res File         25 Note
                4  0 16,   0  2  4,  -1  1  6,   3  3  0,   2  4  6,   3  1  2,
                2  2  2,  -4  0  4,  -1  1  4,   1  7  0,   4  4  0,   1  3  6,
                0  6  3,   2  2  5,   5  1  3,   0  6  1,  -7  1  3,  -8  0  2,
               -1  3  7,  -3  5  3,   0  2  6,  -2  4  1,  -2  4  2,   6  2  1,
               -2  6  2,
PLAT941_ALERT_3_G Average HKL Measurement Multiplicity ..........         3.0 Low
PLAT967_ALERT_5_G Note: Two-Theta Cutoff Value in Embedded .res ..      125.0 Degree
PLAT969_ALERT_5_G The 'Henn et al.' R-Factor-gap value ..........        1.79 Note
              Predicted wR2: Based on SigI**2  5.37 or SHELX Weight 11.18
```

```
PLAT978_ALERT_2_G Number C-C Bonds with Positive Residual Density.         0 Info
```

```
   0 ALERT level A = Most likely a serious problem - resolve or explain
   1 ALERT level B = A potentially serious problem, consider carefully
  28 ALERT level C = Check. Ensure it is not caused by an omission or oversight
  19 ALERT level G = General information/check it is not something unexpected

   0 ALERT type 1 CIF construction/syntax error, inconsistent or missing data
  22 ALERT type 2 Indicator that the structure model may be wrong or deficient
  12 ALERT type 3 Indicator that the structure quality may be low
  11 ALERT type 4 Improvement, methodology, query or suggestion
   3 ALERT type 5 Informative message, check
```

It is advisable to attempt to resolve as many as possible of the alerts in all categories. Often the minor alerts point to easily fixed oversights, errors and omissions in your CIF or refinement strategy, so attention to these fine details can be worthwhile. In order to resolve some of the more serious problems it may be necessary to carry out additional measurements or structure refinements. However, the purpose of your study may justify the reported deviations and the more serious of these should normally be commented upon in the discussion or experimental section of a paper or in the "special_details" fields of the CIF. checkCIF was carefully designed to identify outliers and unusual parameters, but every test has its limitations and alerts that are not important in a particular case may appear. Conversely, the absence of alerts does not guarantee there are no aspects of the results needing attention. It is up to the individual to critically assess their own results and, if necessary, seek expert advice.

Publication of your CIF in IUCr journals

A basic structural check has been run on your CIF. These basic checks will be run on all CIFs submitted for publication in IUCr journals (*Acta Crystallographica*, *Journal of Applied Crystallography*, *Journal of Synchrotron Radiation*); however, if you intend to submit to *Acta Crystallographica Section C* or *E* or *IUCrData*, you should make sure that full publication checks are run on the final version of your CIF prior to submission.

Publication of your CIF in other journals

Please refer to the *Notes for Authors* of the relevant journal for any special instructions relating to CIF submission.

PLATON version of 06/01/2024; check.def file version of 05/01/2024

Datablock 1 - ellipsoid plot

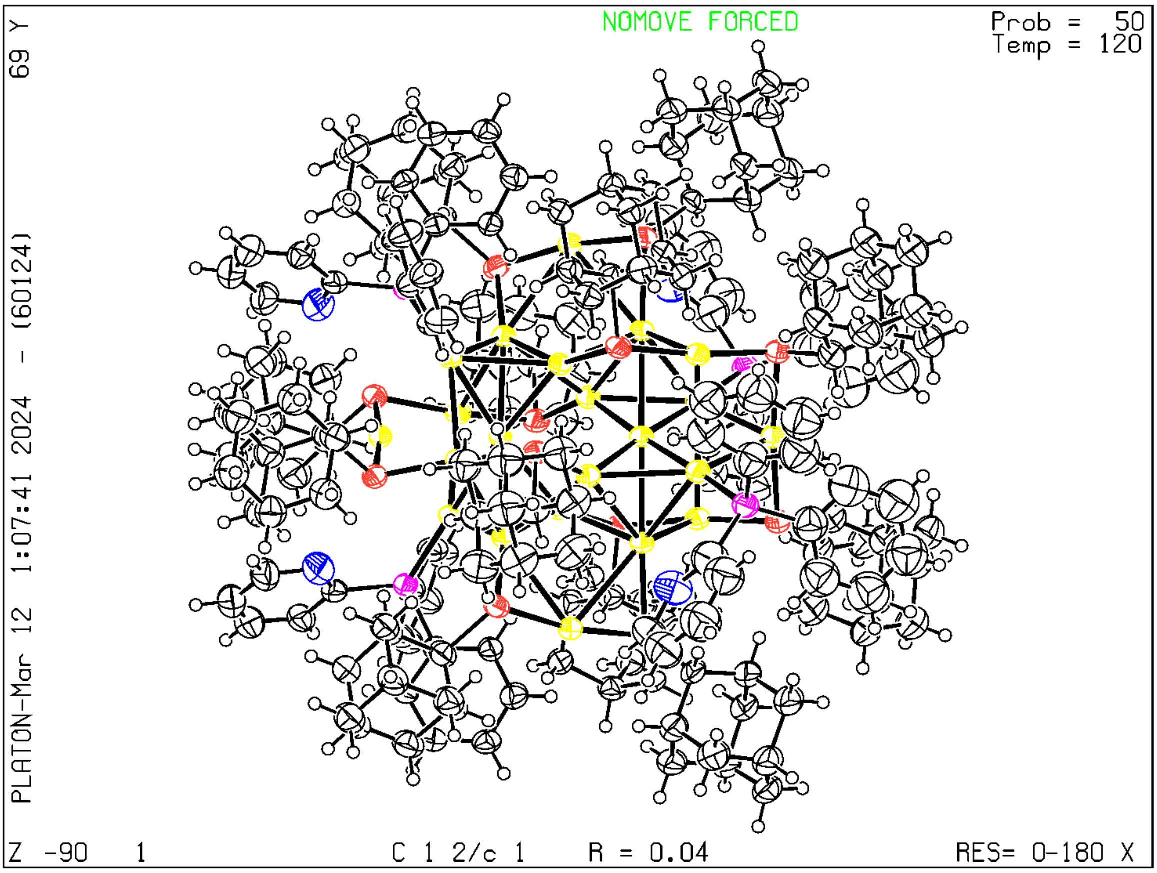

Supplement: Supplementary file 3 — Supplementary Data 1 [file 41467_2025_65024_MOESM3_ESM.zip › Supplementary Data 1/checkCIF Au22.pdf]
